# Adrenal cancer in neurofibromatosis type 1: case report and DNA analysis

**DOI:** 10.1530/EDM-14-0074

**Published:** 2014-11-01

**Authors:** Ravi Kumar Menon, Francesco Ferrau, Tom R Kurzawinski, Gill Rumsby, Alexander Freeman, Zahir Amin, Márta Korbonits, Teng-Teng L L Chung

**Affiliations:** 1 Department of Endocrinology, University College Hospital NHS Foundation Trust, NW1 2PG, London, UK; 2 Centre for Endocrinology, Barts and the London School of Medicine, Queen Mary University of London, EC1A 7BE, London, UK; 3 Department of Endocrine Surgery, University College Hospital NHS Foundation Trust, NW1 2PG, London, UK; 4 Department of Clinical Biochemistry, University College Hospital NHS Foundation Trust, NW1 2PG, London, UK; 5 Department of Pathology, University College Hospital NHS Foundation Trust, NW1 2PG, London, UK; 6 Department of Radiology, University College Hospital NHS Foundation Trust, NW1 2PG, London, UK

## Abstract

Adrenal cortical carcinoma (ACC) has previously only been reported in eight patients with type 1 neurofibromatosis (NF1). There has not been any clear evidence of a causal association between *NF1* gene mutations and adrenocortical malignancy development. We report the case of a 49-year-old female, with no family history of endocrinopathy, who was diagnosed with ACC on the background of NF1, due to a novel germline frame shift mutation (c.5452_5453delAT) in exon 37 of the *NF1* gene. A left adrenal mass was detected by ultrasound and characterised by contrast computerised tomography (CT) scan. Biochemical tests showed mild hypercortisolism and androgen excess. A 24-h urinary steroid profile and ^18^flouro deoxy glucose PET suggested ACC. An open adrenalectomy was performed and histology confirmed ACC. This is the first reported case with DNA analysis, which demonstrated the loss of heterozygosity (LOH) at the *NF1* locus in the adrenal cancer, supporting the hypothesis of an involvement of the *NF1* gene in the pathogenesis of ACC. LOH analysis of the tumour suggests that the loss of neurofibromin in the adrenal cells may lead to tumour formation.

**Learning points:**

- ACC is rare but should be considered in a patient with NF1 and adrenal mass when plasma metanephrines are normal.
- Urinary steroid metabolites and PET/CT are helpful in supporting evidence for ACC.
- The LOH at the *NF1* region of the adrenal tumour supports the role of loss of neurofibromin in the development of ACC.

## Background

Adrenal cortical carcinoma (ACC) is a rare and highly aggressive malignancy. The diagnosis of this condition is challenging and it is often difficult to differentiate between ACC and adrenal adenoma. Urine steroid metabolomics is being used increasingly for both diagnosis and follow-up of people with ACC (1). About 60% of those with ACC have evidence of hormone excess on biochemical analysis. Not all of these are clinically manifested, primarily because of de-differentiation and thus incomplete steroidogenic enzyme expression in malignant cell. This results in increased excretion of steroid precursors in urine. Arlt *et al*. (1) suggested that more than 95% of those with ACC had increased urine excretion of precursor metabolites.

Type 1 neurofibromatosis (NF1) (OMIM 162200) is an autosomal dominant disease with an incidence of one in 3–4000 (2). It is characterised by multiple café-au-lait spots, intertriginous freckling and neurofibromas. Cerebral and spinal tumours, skeletal dysplasias and ophthalmological abnormalities are also found. NF1 is well known to be associated with phaeochromocytoma (3).

ACC is a rare condition, which is associated with several familial cancer susceptibility syndromes, especially Li–Fraumeni, Beckwith–Wiedemann, Gardner and familial adenomatous polyposis coli syndromes (4), as well as multiple endocrine neoplasia type 1. The association with NF1 is not well reported and it is not clear whether this is an incidental finding or if there is a causal association.

## Case presentation

A 49-year-old female of Afro-Caribbean origin with NF1, was under follow-up at the specialist neurofibromatosis clinic in our centre. Genetic analysis in 2011 demonstrated that she had a novel heterozygous mutation c.5452_5453delAT in exon 37 of *NF1* (RefSeq NM_000267.3). In 2012, a routine surveillance ultrasound revealed a left adrenal mass, which on ^123^I-metaIodo benzyl guanidine scan showed weak uptake.

She was referred to our joint endocrine-surgical service and found to have asymptomatic hypertension. There was no family history of endocrine malignancy or endocrinopathy of note. Her blood pressure in clinic was 150/99 mmHg, heart rate 86/min, BMI 31.5 kg/m^2^ and weight 82.5 kg. Clinically, she did not have symptoms or signs suggestive of phaeochromocytoma, glucocorticoid or androgen excess. She had multiple cutaneous neurofibromas, café-au-lait spots, axillary freckling, kyphoscoliosis, and severe myopia. She also had a multi-nodular goitre.

## Investigations

Plasma metanephrines were normal, as was aldosterone/renin ratio. She had mildly elevated androstenedione level at 12.8 nmol/l (1.0–11.8). Her other androgen levels were within normal limits. The low-dose dexamethasone suppression test (LDDST) revealed hypercortisolism excess, with baseline and post-48-h suppression cortisol of 523 and 140 nmol/l respectively. Other pre-operative biochemistry results are available in Table 1.

**Table 1:** Other pre-operative biochemistry

**Pre-operative results**	**Values**	**Normal range**
ACTH	37.1 ng/l	0–46
HbA1c	5.70%	4–6
Urine 17-hydroxypregnanolone	330 μg/24 h	<100

**Figure 1:**
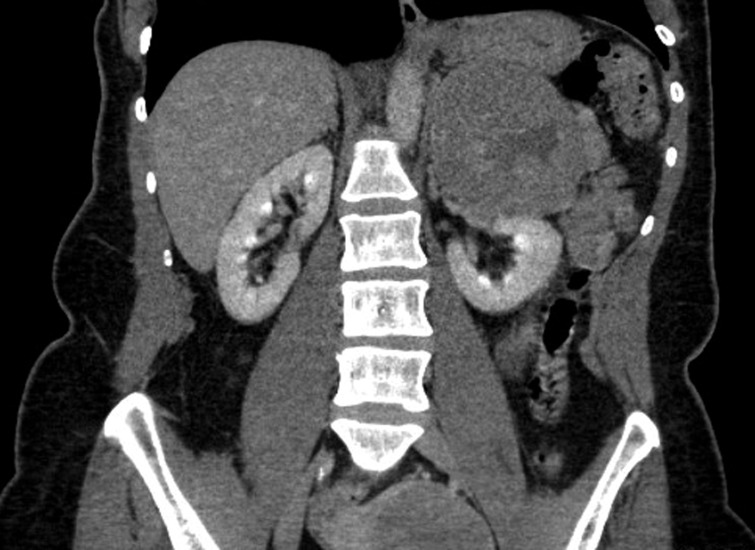
Contrast CT showing a large left-sided adrenal mass with some heterogeneous enhancement and an irregular central cavity probably due to necrosis.

**Figure 2:**
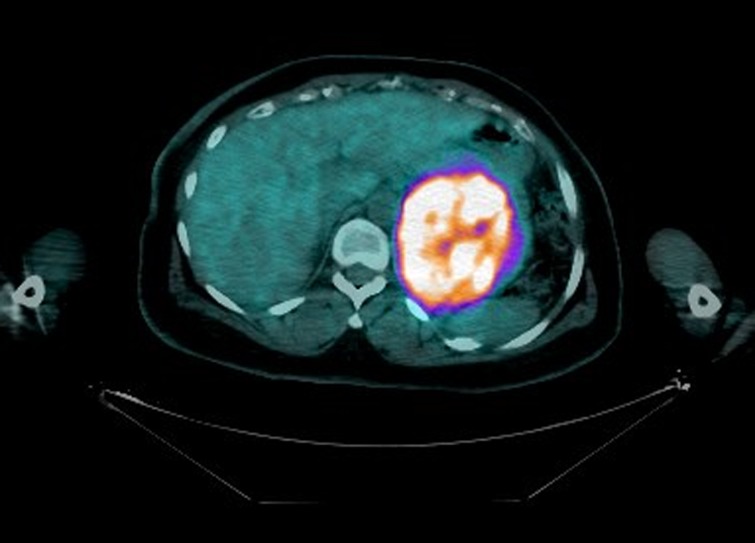
^18^FDG PET showing increased uptake in the left adrenal mass with SUVmax of 25.

Her computerised tomography (CT) scan of the chest and abdomen showed an enhancing 8.7×8.6 cm left supra-renal mass with areas of heterogeneity (Fig. 1). The appearance of a large heterogeneous mass with normal plasma metanephrines and elevated androgen levels gave rise to the suspicion of ACC. We subsequently performed a whole-body ^18^flouro deoxy glucose (FDG) PET/CT, which showed intense increased uptake of ^18^FDG within the mass with a standardised uptake value (SUVmax) of 25, suggesting malignancy (Fig. 2).

**Figure 3:**
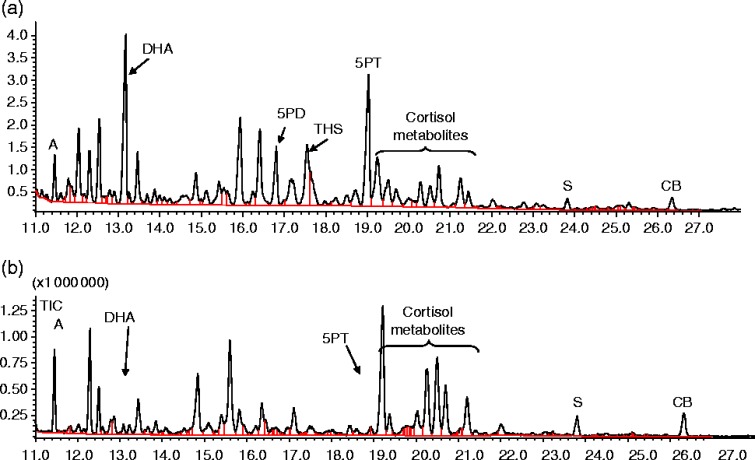
A 24-h urinary steroid profile: (a) pre-adrenalectomy compared with (b) the normal control. The profile is dominated by the Δ5 steroids, in particular pregnenetriol (5PT), DHEA (DHA) and 5PD, and the glucocorticoid precursor, THS. Peaks A, S and CB are internal standards.

A 24-h urinary steroid metabolite profile demonstrated elevated levels of androgen precursors – pregnenediol (5PD), pregnenetriol (5PT) and DHEA (DHA) as well as the glucocorticoid precursor, tetrahydro-11-deoxycortisol (THS) (Fig. 3).

Collating the information, a diagnosis of possible adrenocortical cancer with sub-clinical cortisol and androgen excess was made.

## Treatment

The patient underwent an open adrenalectomy to remove the 9×10 cm adrenal mass. Histology confirmed adrenocortical carcinoma with a modified weiss score of 6. There was no evidence of tumour progression beyond the resected margins. The tumour had areas of confluent necrosis, severe nuclear atypia and increased mitotic activity with focal Ki67 index of 5–10%. Immunohistochemical staining showed calretinin positivity, suggesting cortical origin as opposed to medullary origin. Chromogranin was negative with positive staining for Melan A and inhibin.

## Genetic analysis

The DNA from blood and adrenal tissue were analysed for sequence alterations in the region in which a germline heterozygous AT deletion, c.5452_5453delAT; p.Ile1818Profs*22 in exon 37 of *NF1* (RefSeq NM_000267.3), had been previously found by the Cardiff genetics laboratory.

Germline DNA was extracted from EDTA blood using Illustra DNA extraction kit BACC2 (GE Healthcare, Buckinghamshire, UK) according to the manufacturer's instructions. Tissue DNA extraction from three parallel 5-μm sections of formalin-fixed paraffin-embedded tumour tissue was performed using DNeasy blood and tissue kit (Qiagen) according to the manufacturer's instructions. PCR was performed using forward (5′-ACCTTCATGCACCAGGAGTG-3′) and reverse (5′-ACCGTAAACTGGGTCAGAAC-3′) primers. The primers have been designed using Primer-Blast (www.ncbi.nlm.nih.gov/tools/primer-blast) and synthesised by Sigma–Aldrich.

**Figure 4:**
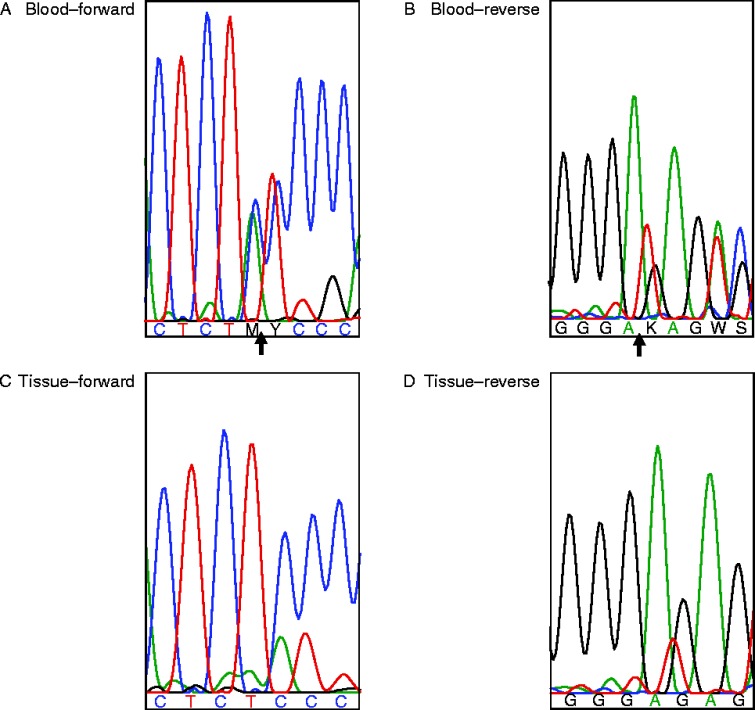
(A) Sequence chromatogram of *NF1* exon 37 in the forward direction, from DNA isolated from peripheral blood. The black arrow indicates the position c.5452_5453 in which an AT heterozygous deletion is identified. In these positions, the software read the heterozygote peaks as M and Y (M=A+C, Y=T+C) because of the presence of only one copy of the WT allele: A-T, which overlaps with C-C following the deletion in the mutated allele. (B) Sequence chromatogram of *NF1* exon 37 in the reverse direction, from DNA isolated from peripheral blood. The reverse sequence is flipped and complementary to the forward one. The black arrow indicates the position c.5452_5453 in which an A-T heterozygous deletion is reported. In these positions, there are two double peaks (A=A+A and K=T+G) because of the presence of only one WT allele, in which A-T is present and overlaps with A-G following the deletion in the mutated allele. (C) Sequence chromatogram of *NF1* exon 37 in the forward direction, from DNA isolated from tumour tissue. The black arrow indicates the position c.5452_5453 in which an A-T hemizygous deletion is reported, confirming loss of heterozygosity. (D) Sequence chromatogram of *NF1* exon 37 in the reverse direction, from DNA isolated from tumour tissue. The reverse sequence is flipped and complementary to the forward one. The black arrow indicates the position c.5452_5453 in which an A-T hemizygous deletion is reported, confirming loss of heterozygosity.

Genetic analysis of the tumour tissue confirmed the loss of the WT allele, therefore suggesting loss of heterozygosity (LOH) at the *NF1* locus (Fig. 4).

## Outcome and follow-up

The post-operative period was uneventful and she did not display any symptoms or signs of adrenal insufficiency (0900 h cortisol 330 nmol/l). On review, 2 months after the surgery, she reported no symptoms. Her blood pressure was 120/75 mmHg (off all anti-hypertensive medications). She had lost 5 kg since surgery. A repeat LDDST showed no evidence of excess hormone secretion. Her repeat 24-h urinary steroid profile showed that the elevated androgenic and glucocorticoid precursor levels had now normalised. A whole-body ^18^FDG PET/CT performed 6 months following the surgery did not show any increased uptake in the tumour bed or elsewhere.

As this was classified as European Network for the Study of Adrenal Tumours (ENSAT) stage 2 ACC, the patient was initiated on adjuvant mitotane 500 mg once daily and the dose has been progressively increased to 5 g daily. Her most recent mitotane level was 12.6 mg/l (target 14–20). She was also started on hydrocortisone when the cortisol level dropped subsequent to starting mitotane. She has remained in remission 18 months after the surgery.

## Discussion

We describe an unusual case of ACC in a patient with NF1. This is the ninth case of ACC reported in the NF1 in the literature and the first case that demonstrated the LOH at the *NF1* locus in ACC.

NF1 results from a loss-of-function mutation or deletion in the *NF1* gene located on chromosome 17. *NF1* is a tumour suppressor gene encoding neurofibromin. Patients are susceptible to a variety of tumours, of which the most common malignant tumours are sarcomas (including leiomyosarcoma and neurofibrosarcoma), and carcinoma of the breast, lung and GI tract. Of the other tumours, gastro-intestinal stromal tumours, optic nerve gliomas, phaeochromocytomas, parathyroid adenomas, carcinoid tumours and thyroid nodules are also common (5).

Neurofibromin is widely expressed in humans at the earliest stages of development, while in adults it is expressed mostly in neural tissues (neurons, Schwann cells and oligodendrocytes) and in the adrenal medulla. Neurofibromin acts as a GTPase activator and is a negative regulator of the Ras oncoprotein involved in cell differentiation and proliferation that requires GTP for full activity (6).

In patients with a heterozygous germline *NF1* mutation, the loss of the other (WT) allele will lead to the complete loss of neurofibromin function and the development of tumours, according to the Knudson two-hit hypothesis (7). This LOH at the *NF1* locus has been found in both benign and malignant tumours associated with germline *NF1* mutations. Interestingly, somatic *NF1* mutations are common in sporadic phaeochromocytomas (8).

ACC has been reported in NF1. A literature search yielded five confirmed and three more possible cases of ACC in NF1. In 1967, Fraumeni & Miller (9) reported a case of NF1 associated with ACC in a 4.5-year-old girl who also had brain metastasis. In the report, there is also mention of two other cases (16- and 17-year-old girls) of ACC associated with café-au-lait spots. However, other than café-au-lait spots, no other features of NF1 were described and there are no follow-up data. In 1970, Fienman & Yakovac (10) reported one case of neurofibromatosis and adrenal carcinoma with a thalamic tumour, but the report did not have any further details. Sorensen *et al*. (11) followed up a cohort of Danish patients with NF1 who were identified 42 years previously and reported two cases of ACC in a series of 212 malignant tumours in patients with NF1. One was a 46-year-old female who also had reticulosarcoma and there are no clinical details on the second case. In 2005, Wagner *et al*. (12) reported the case of a 3-year-old girl with NF1 and ACC. She had a paraspinal metastasis and presented with the clinical features of cortisol and androgen excess. The last case is from Gutmann *et al*. (13) in which they found a LOH for the 17q region in the adrenal tumour in their patient with NF1.

Among the previously reported cases of ACC in NF1, in only one case has there been a loss-of-heterozygosity analysis performed on the tissue sample from the adrenal cortical tumour, it is not clear whether this was an ACC (13). Our analysis suggests that the LOH in the tumour may have a direct link with the development of her ACC.

In conclusion, we report herein an NF1 patient with a novel *NF1* gene mutation and an ACC. LOH analysis of the tumour suggests that the loss of neurofibromin in the adrenal cells may lead to tumour formation.

## Patient consent

The authors confirm that written informed consent was obtained from the patient for publication of the submitted article and the accompanying images.

## Author contribution statement

R K Menon and T-T L L Chung contributed to literature search and writing of the manuscript. F Ferrau and M Korbonitis provided the DNA analysis report and contributed to the writing of the manuscript. All other co-authors contributed towards patient care and finalising the draft.
